# Therapeutic Dosage of Antipsychotic Drug Aripiprazole Induces Persistent Mitochondrial Hyperpolarisation, Moderate Oxidative Stress in Liver Cells, and Haemolysis

**DOI:** 10.3390/antiox12111930

**Published:** 2023-10-30

**Authors:** Tinkara Pirc Marolt, Barbara Kramar, Andrej Vovk, Helena Podgornik, Dušan Šuput, Irina Milisav

**Affiliations:** 1 Institute of Pathophysiology, Faculty of Medicine, University of Ljubljana, Zaloska 4, 1000 Ljubljana, Slovenia; 2 Department of Haematology, University Medical Centre Ljubljana, 1000 Ljubljana, Slovenia; 3 Faculty of Pharmacy, University of Ljubljana, Aškerčeva cesta 7, 1000 Ljubljana, Slovenia; 4 Laboratory of Oxidative Stress Research, Faculty of Health Sciences, University of Ljubljana, Zdravstvena pot 5, 1000 Ljubljana, Slovenia

**Keywords:** mitochondria, hyperpolarisation, aripiprazole, cell respiration, schizophrenia, olanzapine, hepatocytes, drug-induced liver injury

## Abstract

Aripiprazole has fewer metabolic side effects than other antipsychotics; however, there are some severe ones in the liver, leading to drug-induced liver injury. Repeated treatment with aripiprazole affects cell division. Since this process requires a lot of energy, we decided to investigate the impact of aripiprazole on rat liver cells and mitochondria as the main source of cellular energy production by measuring the mitochondrial membrane potential, respiration, adenosine triphosphate (ATP) production, oxidative stress, antioxidative response, and human blood haemolysis. Here, we report that mitochondrial hyperpolarisation from aripiprazole treatment is accompanied by higher reactive oxygen species (ROS) production and increased antioxidative response. Lower mitochondrial and increased glycolytic ATP synthesis demand more glucose through glycolysis for equal ATP production and may change the partition between the glycolysis and pentose phosphate pathway in the liver. The uniform low amounts of the haemolysis of erythrocytes in the presence of aripiprazole in 25 individuals indicate lower quantities of the reduced form of nicotinamide adenine dinucleotide phosphate (NADPH+H^+^), which is in accordance with a decreased activity of glucose 6-phosphate dehydrogenase and the lower dehydrogenase activity upon aripiprazole treatment. The lower activity of glucose 6-phosphate dehydrogenase supports a shift to glycolysis, thus rescuing the decreased mitochondrial ATP synthesis. The putative reduction in NADPH+H^+^ did not seem to affect the oxidised-to-reduced glutathione ratio, as it remained equal to that in the untreated cells. The effect of aripiprazole on glutathione reduction is likely through direct binding, thus reducing its total amount. As a consequence, the low haemolysis of human erythrocytes was observed. Aripiprazole causes moderate perturbations in metabolism, possibly with one defect rescuing the other. The result of the increased antioxidant enzyme activity upon treatment with aripiprazole is increased resilience to oxidative stress, which makes it an effective drug for schizophrenia in which oxidative stress is constantly present because of disease and treatment.

## 1. Introduction

Aripiprazole (ARI) is an antipsychotic drug for treating mental disorders such as schizophrenia and bipolar disorder. It has fewer side effects than other frequently used second-generation antipsychotics [1]. Nevertheless, the adverse effects include impulsive and compulsive behaviours, such as uncontrollable urges to gamble, overeat, or shop [2]. The metabolic side effects include diabetes mellitus [3], rhabdomyolysis [4], hypertension [5], elevated liver enzymes [4], and even cases of life-threatening complications, like drug-induced liver injury (DILI) [6,7,8]. DILI associated with antidepressant use is mostly hepatocellular [8], which fits with a reduced proliferative capacity of repeated ARI-treated liver cells in vitro [9]. Liver cell survival, on the other hand, is not affected by 6 mM ARI concentrations in single or up to 8 weeks of daily treated cells [9]; also, no differences in survival or function of macrophage-like RAW264.7 cells were reported after a single 10 mM ARI treatment and C6 glioma cells survival equalled that of untreated controls when exposed to single ARI concentrations of up to 100 mM [10]. Unchanged cell survival of the macrophage-like and glioma cells was inferred from their dehydrogenase activities (3-(4,5-dimethylthiazol-2-yl)-2,5-diphenyl-2H-tetrazolium bromide, MTT test). While the survival of the glioma cells is in accordance with that of the liver cells, the unchanged dehydrogenase activities in glioma cells are in sharp contrast with statistically significantly reduced dehydrogenase activities even after a single addition of 2.23 mM ARI in liver cells [9]. Therefore, evaluating the effects of ARI in different cell types and tissues, among them in the liver, is needed, as ARI metabolism is predominantly hepatic [11].

Mitochondrial dysfunction was linked to the adverse effects of antipsychotic drugs [12,13]. Some first-generation medicines—chlorpromazine, fluphenazine, and haloperidol—as well as second-generation—clozapine and risperidone—inhibited the activity of complex I in the brains of schizophrenia patients [14]. Haloperidol also decreased complex I activity in chronically fed rats’ brains, while clozapine did not [15,16]. Measurements of oxygen consumption in isolated mitochondria detected a decrease in electron transfer in some second-generation antipsychotics, such as quetiapine, but not others, e.g., olanzapine (OLA) [17]. Contradictory influences on the respiratory chain of antipsychotics such as ARI and OLA have been reported: lower activity and expression of the respiratory chain proteins in lymphoblastoid cells exposed to OLA [18] but not to ARI. On the other hand, OLA did not affect mitochondrial respiration, while ARI inhibited respiratory complexes in mitochondria from pig brains [19]. A decrease in intracellular adenosine triphosphate (ATP) in neuronal PC12 cells was reported with ARI but not OLA [20]. The aerobic and glycolytic metabolism of microglial cells BV-2 were affected by ARI [21]. ARI had a stimulatory effect on pyruvate dehydrogenase, citrate synthase, and complex I in PC12 cells, while it suppressed cytochrome c oxidase (complex IV) activity [22]. This phenomenon was observed by large concentrations of ARI (50 mM) and very high glucose concentrations (25 mM). The authors also reported an increased mitochondrial membrane potential, i.e., mitochondrial hyperpolarisation.

Transient mitochondrial hyperpolarisation was reported in normal T cells as an immediate consequence of T-cell receptor stimulation, followed by transient mitochondrial membrane depolarisation and a spike in the ATP concentration [23]. A reversible mitochondrial hyperpolarisation was thus accompanied by an induction of glycolysis for energy production and activation of the pentose phosphate pathway [23]. An increased membrane potential also develops within two minutes of creating a gap in the bladder epithelial cell sheet in cell cultures and lasts until a continuous cell sheet is reformed [24]. There are more reports on short mitochondrial hyperpolarisation, mainly in T cells, as an effect leading to apoptosis [23,25,26,27] and ferroptosis, an iron-dependent form of regulated necrosis in an experimental setting [28]. The increased mitochondrial membrane potential is also reported in T cells of type 1 diabetes mellitus patients but not type 2 [23]. This hyperpolarisation is not associated with glycolytic changes and lasts longer; therefore, it seems distinct from activation-induced reversible mitochondrial hyperpolarisation of the normal T cells described above. The transient membrane potential elevation and the resultant ATP depletion sensitise T cells in systemic lupus erythematosus patients to necrosis, thus contributing to increased inflammation [29,30]. However, the causes of hyperpolarisation differ, at least between patients with type 1 diabetes and systemic lupus erythematosus [23]. Mitochondria are also hyperpolarised in many cancer cells; from 200 cell lines/types, derived from tumours from 17 organs, including the liver, most adenocarcinoma, melanoma, squamous cell carcinoma, and transitional cell carcinoma had higher membrane potential than noncancer cells [31]. In breast and colonic carcinoma cells, differences in the membrane potential reflect the cell heterogeneity [32]. The invasiveness of the colonic carcinoma cells increases with the mitochondrial hyperpolarisation, as cells with a higher mitochondrial membrane potential have an enhanced capacity to respond to hypoxia, grow under anchorage-independent conditions, and can invade the basement membrane [33].

Increased oxidative stress occurs as a consequence of mitochondrial hyperpolarisation [34,35,36]. Oxidative stress is an important factor in psychiatric disease development, including schizophrenia [37]. Many antipsychotics can protect against oxidative stress [38,39,40]. Increased survival and higher superoxide dismutase expression under oxidative stress were detected in ARI- and OLA-treated PC12 neuronal model cells compared to the untreated controls [39,41]. ARI protects liver cells only during oxidative stress by increased activity of antioxidant enzymes and upregulation of proteins associated with oxidative stress response [40] and is bound by glutathione (GSH) [42]. Without an oxidant, ARI does not affect cell death or induce apoptosis, still reducing the rate of hepatocytes’ division [9].

Since cell division requires a lot of energy, we decided to investigate the impact of ARI on mitochondria as the main source of cellular energy production. Our hypothesis was that ARI reduces mitochondrial energy production. Here, we report on the immediate mitochondrial hyperpolarisation in ARI-treated cells and isolated mitochondria, which is persistent and does not lead to apoptosis. ATP-linked oxygen consumption and mitochondrial ATP production are reduced upon ARI treatment, which cells compensate for by increased glycolysis. The amount of ROS increases possibly because of mitochondrial hyperpolarisation. Higher amounts of ROS increase the activities of antioxidative enzymes, resulting in cell survival. The amount of total glutathione is reduced, likely because of the ARI-GSH binding. The effect of lower GSH is observed in the ARI-induced haemolysis of human erythrocytes.

## 2. Materials and Methods

All reagents were purchased from Sigma-Aldrich (Merck, St. Louis, MO, USA) unless otherwise stated.

### 2.1. Cell Culture

The rat hepatoma cell line Fao (ECACC, 89042701) was grown and treated with ARI (PHR1784, CAS Number: 129722-12-9) or OLA (PHR1825, CAS Number: 132539-06-1) as previously described [9]. Several parallels from different batches of cells were treated daily with ARI, OLA, or 0.12% dimethyl sulfoxide (DMSO), as the vehicle control (untreated control), for between four and eight weeks when used in the experiments [9].

Ninety-six-well, flat-bottom transparent and black culture plates were used for the hydrogen peroxide (H_2_O_2_) measurements and a microplate-based (tetramethylrhodamine methyl ester) TMRM assay, respectively. Cells were seeded at 4 × 10^4^ cells/well, and 2.4 × 10^6^ cells/flask were seeded in T25 flasks to evaluate the activities of antioxidant enzymes and glutathione content. Twelve-well plates (4 × 10^5^ cells/well) were used for microcapillary flow cytometry, and 15 mm collagen-coated coverslips (1 × 10^5^ cells/coverslip) for microscopic observations (mitochondrial membrane potential). For the mitochondria isolation from Fao cells, 7.2 × 10^6^ cells were seeded in T75 flasks, and 24-well plates provided by the supplier (4.5 × 10^4^ cells/well) were used in Agilent Seahorse XFe24 Analyser.

Hepatocytes were isolated (ethical code number U34401-44/2014/8, issued by Administration of the Republic of Slovenia for Food Safety, Veterinary Sector, and Plant Protection) and grown as described by Miller et al. [43]. Approximately 3 × 10^4^ cells were seeded in 96-well, flat-bottom black culture plates for the TMRM assay. Hepatocytes were treated with ARI for 48 h, starting 24 h after the seeding.

### 2.2. Mitochondrial Isolation

Mitochondria were isolated from the liver of adult Wistar–Hannover rats (16 weeks) donated by the Medical Experimental Centre (re-use, Faculty of Medicine, University of Ljubljana, ethical code number U34401-17/2016/3, issued by Administration of the Republic of Slovenia for Food Safety, Veterinary Sector and Plant Protection). Centrifugation was performed at 4 °C (Sigma 3K30), and all other steps were completed on ice. The liver was cut into small pieces and washed with sucrose-EDTA (SE) buffer composed of 250 mM sucrose, 10 mM 4-(2-hydroxyethyl)-1-piperazineethanesulfonic acid (HEPES), and 2 mM ethylenediaminetetraacetic acid (EDTA). One gram of washed liver pieces was diluted in 10 mL buffer containing 10 μL/mL protease inhibitor cocktail and 1 mM phenylmethylsulfonyl fluoride (PMSF). Liver pieces were homogenised using a Potter–Elvehjem homogeniser and centrifuged for 10 min at 700× *g*. The supernatant, after the third homogenisation, was spun for 10 min at 6800× *g*, and then the pellet was washed in 10 mL buffer and centrifuged again for 10 min at 6800× *g*. Finally, the pellet was resuspended in 0.5 mL of SE buffer, aliquoted, frozen in liquid nitrogen, and stored at −70 °C.

Mitochondria were also isolated from long-term treated Fao cells. For each treatment, cells were collected from two T75 flasks in 1 mL phosphate-buffered saline (PBS) using a rubber cell scraper and spun for 10 min at 700× *g*. After discarding the supernatant, further steps were performed, as described above, for the rat liver mitochondria, only in 20× smaller volumes. The final pellets were resuspended in 50 μL buffer.

### 2.3. Protein Content

The protein content was determined with BCA Pierce (Thermo Fischer Scientific, Waltham, MA, USA). The absorbance was measured with a microplate reader (Victor 1420-050, PerkinElmer, Waltham, MA, USA).

### 2.4. Mitochondrial Membrane Potential Measurement in the Cells

The mitochondrial membrane potential was evaluated with 1 μM or 150 nM tetramethylrhodamine methyl ester (TMRM). For the microplate assay, 10 μL of 10 mM TMRM was added for 100 μL assay per well in growth media. Cells were washed once with 100 μL PBS buffer for 10 min after 30 min of incubation at 37 °C. Then, the fluorescence was measured in 100 μL PBS using appropriate filter sets (ex. 535/20 nm, em. 595/20 nm, Synergy H4, BioTek Instruments, Winooski, VT, USA).

To visualise the TMRM’s accumulation in the mitochondria, cells were stained with 10 μL of 1.5 mM TMRM stock solution per 1000 mL of growth media for 15 min. After staining, the coverslip was fixed in a holder, covered with 200 μL growth media, and observed with an Olympus IX81F inverted fluorescent microscope (ex. 530–550 nm, em. 590 nm).

### 2.5. Mitochondrial Membrane Potential Measurement in Isolated Mitochondria

Isolated rat liver mitochondria were quickly thawed and immediately placed on ice until further use. Then, 150 nM TMRM was prepared in media containing 3% fatty acid-free bovine serum albumin (BSA), 250 mM sucrose, 80 mM KCl, 20 mM HEPES (KOH pH 7.2), 5 mM MgCl_2_, 2 mM ATP, 2 mM nicotinamide adenine dinucleotide phosphate (NADPH+H^+^), 2.5 mM malate, and 2.5 mM succinate. Mitochondria were added at a concentration of 0.5 mg protein per mL, treated with 6 μM ARI for 5 min, and observed with a super-resolution confocal inverted microscope from ZEISS LSM 900 (ex. 530 nm, em. 590 nm). Images were analysed online with MATLAB version R2021a (DOI:10.5281/zenodo.7950424). The Wiener2—pixel-wise adaptive low-pass filter function was used to remove the background noise from the imported grayscale microscopic images. The neighbourhood of the filtering was set to 5 by 5 pixels. Segmentation of the image started by calculating the gradient of an image and applying the threshold. The threshold was defined with the usage of the edge and Sobel operators. A dilation operation was performed to connect the gradient lines that partially outlined the object. The filling of the interior gaps of objects was achieved with the infill function, and, finally, the outline of the objects was smoothed by eroding the image with the diamond structural element. Some small structures below 2 pixels were additionally removed with the imopen function. The mean values of those segmented regions were computed. The threshold of half of the max intensity in the image was used to delineate the brighter areas at some locations. Then, the ratio of the brighter areas over the segmented areas with the signal was also calculated. The final values are presented as the percentage of the fluorescent signal (i.e., the difference between the fluorescence before and after the FCCP treatment divided by the fluorescence before the FCCP treatment).

### 2.6. Mitochondrial Metabolic Function and Mitochondrial Complex Activity

The Agilent Seahorse XFe24 Analyser was used to monitor the oxygen consumption rate (OCR) and extracellular acidification rate (ECAR). The mitochondrial function was evaluated using the Agilent Seahorse XF Cell Mito Stress Test (Agilent Technologies, Santa Clara, CA, USA, 103015-100) with XF RPMI Medium (Agilent Technologies, 103576-100) according to the manufacturer’s instructions. We used 1.5 μM oligomycin, 1 μM FCCP, 0.5 μM rotenone, and 0.5 μM antimycin as modulators of respiration to monitor the critical parameters of the mitochondrial function, including basal and maximal respiration, proton leak, coupling efficiency, nonmitochondrial respiration, and spare respiratory capacity. The mitochondrial and glycolytic ATP production rates, as well as the total ATP production rate, were determined as described by Lund and coworkers [44] and Romero and coworkers [45].

For measuring the individual mitochondrial complex activity, the same kit was used with the mitochondrial assay solution buffer (Agilent Technologies) in combination with 1 nM XF Plasma Membrane Permeabilizer (Agilent Technologies) and relevant substrates, including 10 mM pyruvate, 10 mM glutamate, 10 mM succinate, and 10 mM ascorbate, following the manufacturer’s recommendations.

The activity of complex V was evaluated with the MitoCheck Complex V Activity Assay Kit (Cayman Chemical, Ann Arbor, MI, USA) using isolated rat liver and long-term treated Fao mitochondria. It measures ATPase activity through coupled reactions of substrate-level phosphorylation of adenosine diphosphate (ADP) to ATP by pyruvate kinase upon the conversion of phosphoenolpyruvate to pyruvate and the subsequent conversion of pyruvate to lactate by lactate dehydrogenase and nicotinamide adenine dinucleotide (NADH) oxidation. The formation of NAD+ is measured at 340 nm. Measurements were obtained using the Epoch Microplate Spectrophotometer (BioTek Instruments).

### 2.7. ATP Content

For the total ATP levels in cell lysates, samples were prepared and measured using the ATP Detection Assay Kit—Luminescence (Cayman Chemical) according to the manufacturer’s recommendations. A luminescent signal was detected with the Epoch Microplate Spectrophotometer (BioTek Instruments).

### 2.8. Oxidative Stress (Superoxide Measurement)

The Muse Oxidative Stress Kit was used, which mainly measures superoxide (MCH100111, Merck, Rahway, NJ, USA). Cells were diluted in assay buffer at 10^6^ cells/mL, then incubated with Muse Oxidative Stress Reagent for 30 min, and analysed with Guava Muse Cell Analyser (Luminex, Austin, YX, USA).

### 2.9. H_2_O_2_ Production

The H_2_O_2_ production was measured using the Amplex Red Hydrogen Peroxide/Peroxidase Assay Kit (A22188, Invitrogen, Molecular Probes, Eugene, OR, USA) according to the manufacturer’s recommendations. Absorbance was measured in microplates using a Victor reader (1420-050, PerkinElmer).

### 2.10. Antioxidant Enzymes and Glutathione Measurements

The activities of catalase, superoxide dismutase, glutathione peroxidase, and glutathione content were determined with the relevant Cayman Chemical Assay Kits (707002, 706002, 703102, and 703002) following the manufacturer’s instructions. The measurements were acquired with a Victor microplate reader (1420-050, PerkinElmer) or an Epoch Microplate Spectrophotometer (BioTek Instruments).

### 2.11. Haemolysis Assay

The study complied with the Helsinki Declaration and was approved by the Slovenian Ethics Committee (KME43/02/09). Residual blood without a donor’s identifier was obtained from 25 donors (9 males, 16 females) with normal complete blood counts in K_2_-EDTA-coated vacutainer tubes. The protocol was adapted from Evans et al. [46]. The blood was centrifuged at 1000× *g* for 15 min, and 250 μL of erythrocytes (bottom layer) was washed twice with 250 μL saline buffer containing 150 mM NaCl and 10 mM HEPES (pH 7.4). Two centrifugation steps were performed at 1000× *g* for 5 min between the washing steps. After the washes, erythrocytes were resuspended in 250 μL of saline buffer and diluted 50x with saline buffer. For the haemolysis assay, 50 μL of 20× stock solution of DMSO, ARI, or OLA was added to a 1.5 mL microcentrifuge tube and mixed with 950 μL of erythrocyte suspension and incubated for 2 h at 37 °C. For a positive control, 1% Triton X-100 was used. After incubation, the samples were centrifuged at 2000× *g* for 5 min. Then, 200 μL of supernatant was loaded on transparent 96-well plates in two parallels. The absorbance at 415 nm was measured with an Epoch Microplate Spectrophotometer (BioTek Instruments).

### 2.12. Statistical Analysis

The statistical analysis was performed with GraphPad Prism 9.2.0 unless otherwise stated. All data are presented as the means ± standard deviation (SD). Data with more than 2 groups were analysed with one-way analysis of variance (ANOVA). The number of biological replicates (n) is noted in the figure legends. Correction for multiple comparisons was performed using Dunnett’s method to indicate statistical significance between untreated controls and treatments.

## 3. Results

The concentrations of ARI that were reported for the patients’ plasma were chosen to closely model the effects of ARI on the patient’s liver cells, namely, a therapeutic concentration (0.3 μM) and the concentration with an increased risk of drug toxicity (laboratory alert: 2.23 μM) [47]. A higher, 6 μM concentration was used, as the liver concentration of some drugs is higher than that of plasma [48]. The steady-state levels of ARI in the human body that are reached after two weeks of treatment [49] were mimicked by treating the liver hepatoma Fao cells for four to eight weeks in addition to measuring their acute response after a single 24 h period of ARI exposure.

**Figure 1:**
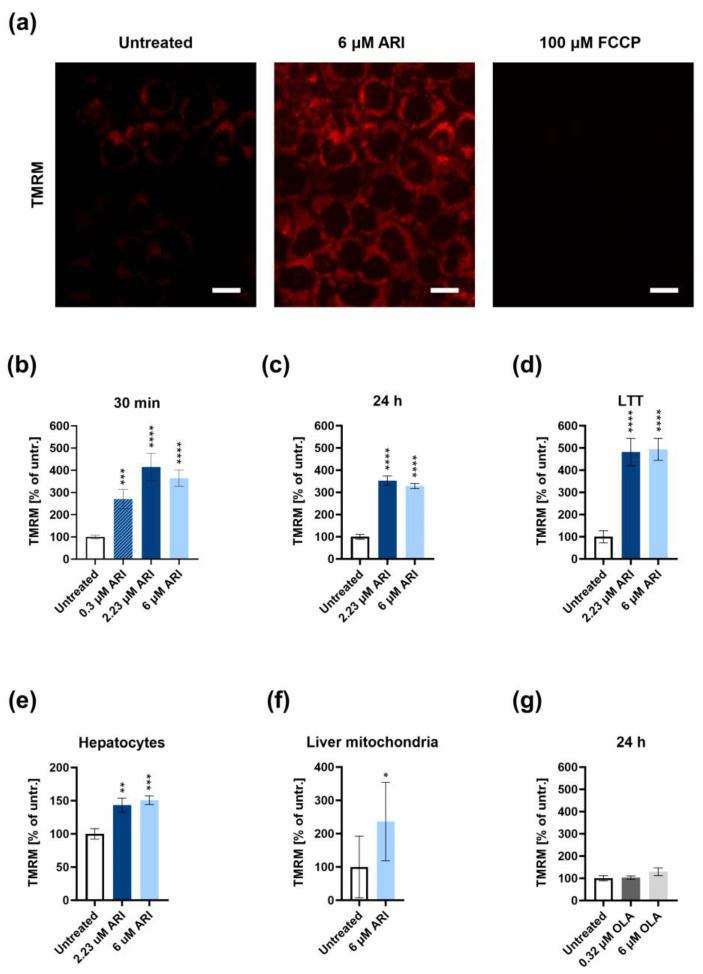
Mitochondrial hyperpolarisation of liver cells and isolated mitochondria. (**a**) Representative confocal images of successively treated tetramethylrhodamine methyl ester (TMRM) labelled Fao cells: (**left**) untreated; (**middle**) ARI-treated for 5 min; after the addition of the uncoupler (carbonyl cyanide-p-trifluoromethoxyphenylhydrazone, FCCP). Scale bar: 10 μm. Mitochondrial membrane potential was measured spectrophotometrically in the Fao cells with TMRM after the ARI treatment for (**b**) 30 min, (**c**) 24 h, and (**d**) 4–6 weeks (long-term treatment, LTT). All experiments were conducted in four biological replicates (*n* = 4). Mitochondrial membrane potential: (**e**) ARI-treated hepatocytes for 48 h (*n* = 3); (**f**) isolated liver mitochondria (*n* = 7; see also Supplementary Materials Figure S1b). (**g**) Spectrophotometric measurement of the mitochondrial membrane potential of olanzapine (OLA)-treated cells (*n* = 3). Data are presented as the mean ± SD and analysed with one-way ANOVA followed by Dunnett’s test, except for the isolated liver mitochondria, where the two groups were compared with a *t*-test. * *p* ≤ 0.05, ** *p* ≤ 0.01, *** *p* ≤ 0.001, and **** *p* ≤ 0.0001. untr.: untreated.

Hyperpolarisation of the mitochondria was observed in the ARI-treated Fao cells, hepatocytes, and isolated liver mitochondria (Figure 1). Elevation of the mitochondrial membrane potential was already detected after 5 min of ARI treatment in the Fao cells and liver mitochondria. Mitochondria were hyperpolarised when treated with physiological concentrations of ARI (Figure 1b). The hyperpolarisation was instant after the ARI addition, and the membrane potential remained equal or even slightly higher during continuous treatment for 1–2 months (Figure 1d). This effect was ARI specific, as adding another second-generation antipsychotic, OLA, did not affect the mitochondrial membrane potential (Figure 1g).

**Figure 2:**
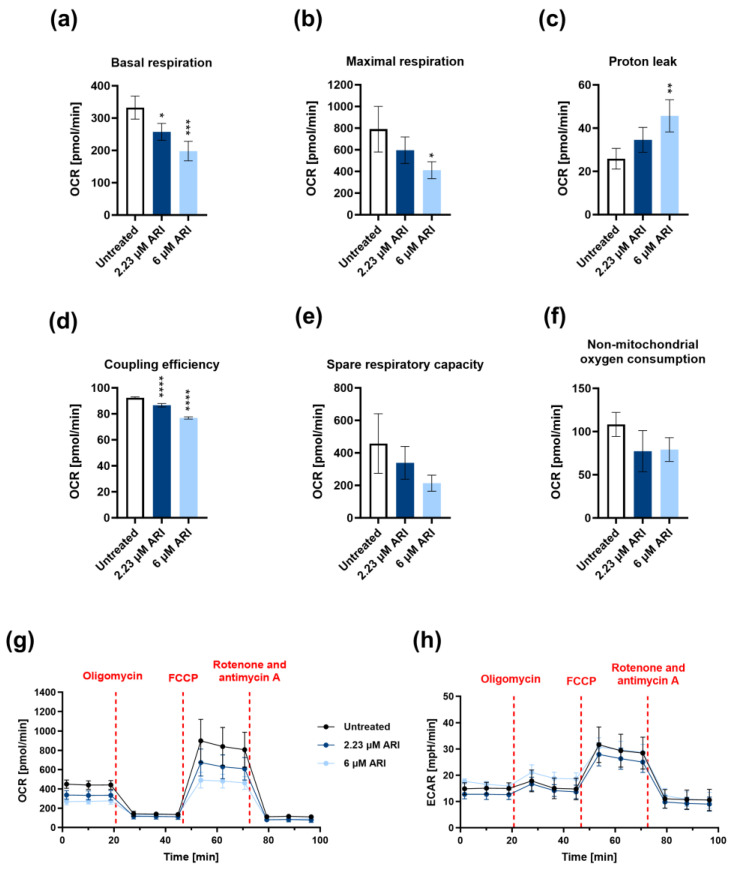
Mitochondrial respiration of ARI-treated Fao cells for 24 h: (**a**) basal respiration; (**b**) maximal respiration; (**c**) proton leak; (**d**) coupling efficiency; (**e**) spare respiratory capacity; (**f**) nonmitochondrial oxygen consumption; (**g**) oxygen consumption rate (OCR) values; (**h**) extracellular acidification rate (ECAR) values. Data from four biological replicates (*n* = 4) are presented as the mean ± SD and analysed with one-way ANOVA followed by Dunnett’s test. * *p* ≤0.05, ** *p* ≤ 0.01, *** *p* ≤ 0.001, and **** *p* ≤ 0.0001.

The cells were not apoptotic despite the hyperpolarisation [9]. Therefore, we looked at their mitochondrial function. There was a significant decrease in the overall respiration after 24 h of ARI treatment (Figure 2). The ARI-treated cells had a statistically significant lower basal respiration, i.e., lower mitochondrial ATP production under basal conditions (Figure 2a). The ability of the respiratory chain to operate at the maximum capacity and maximal respiration was also significantly reduced at 6 mM ARI treatment (Figure 2b). Under these conditions, the proton leak significantly increased (Figure 2c). The proton leak is a part of the basal respiration not coupled to ATP production and can indicate mitochondrial damage. The proportion of the basal respiration that drives ATP synthesis (the ATP-linked oxygen consumption) was significantly reduced in all ARI-treated cells after 24 h (coupling efficiency, Figure 2d). There was also a trend of a reduced spare respiratory capacity, which is the cell’s ability to increase respiration in cases of higher demand (Figure 2e). This reduction was clearly seen in each biological replicate. There was also a trend of reduced oxygen use by nonmitochondrial enzymes (Figure 2f, nonmitochondrial oxygen consumption).

**Figure 3:**
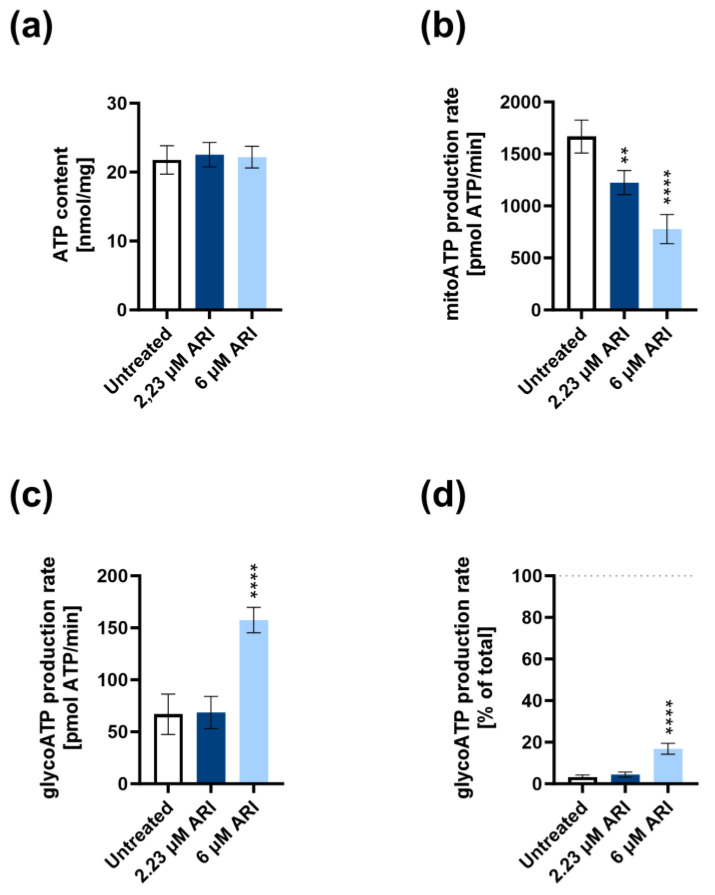
ATP production in Fao cells treated with or without ARI for 24 h: (**a**) total cellular ATP was measured with a luminescent assay (*n* = 4); (**b**) mitochondrial and (**c**) glycolytic ATP production rates were calculated from respirometry measurements (*n* = 4); (**d**) glycolytic ATP production rate expressed as a percentage of the overall ATP production rate. Data are presented as the mean ± SD and analysed with one-way ANOVA followed by Dunnett’s test. ** *p* ≤ 0.01 **** *p* ≤ 0.0001.

Although overall ATP content did not differ between the ARI-treated and untreated cells (Figure 3a), the mitochondrial ATP production rate was significantly reduced by both ARI doses in the 24 h-treated Fao cells (Figure 3b). A significant glycolytic ATP production rate increase of up to 20% of the total ATP production was observed in the 6 μM ARI-treated cells (Figure 3c,d). This decrease was ARI specific, as it did not occur in the OLA-treated cells (Supplementary Materials Figure S2).

**Figure 4:**
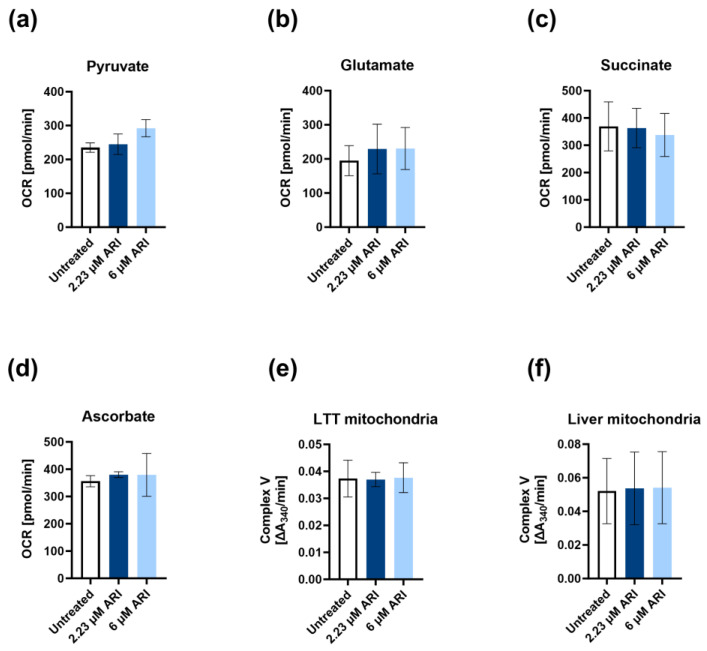
Contribution of individual mitochondrial complexes to respiration and ATP production. The oxygen consumption rate (maximal respiration) for the respective substrates: (**a**) pyruvate; (**b**) glutamate; (**c**) succinate; (**d**) ascorbate measured in permeabilised Fao cells. Activity of complex V (ATP synthase): (**e**) mitochondria derived from long-term treated (LTT) Fao cells; (**f**) isolated rat liver mitochondria. All experiments were performed in three biological replicates (*n* = 3). OCR: oxygen consumption rate. Data are presented as the mean ± SD and analysed with one-way ANOVA.

Further assessments of the role of ARI on oxidative phosphorylation by measuring the respiration of specific substrates, pyruvate, glutamate (complex I), succinate (complex II), and ascorbate (complex IV) did not reveal differences in the mitochondrial metabolic activities. There was no difference in the maximal respiration in permeabilised Fao cells between the ARI-treated and control cells (Figure 4a–d). Consistent, although statistically not significant, reductions in the oxygen consumption rates were observed for individually added substrates (pyruvate, glutamate, and succinate) before oligomycin was injected (Supplementary Materials Figure S3), implying decreased function of complex V (ATP synthase). No difference was detected in the ATP synthesis rate measured by the equimolar oxidation of NADH into NAD^+^. As the ATP synthase activity methods used here and from other providers measure only the reverse ATP synthase activity, we proved that conversion of ATP to ADP was unaffected by the ARI treatment in long-term treated Fao and liver mitochondria (Figure 4e,f). As the statistically significant decrease in the mitochondrial ATP production rate was measured by the respirometry, ARI-affected complex V (ATP synthase) activity and, in this way, at least partially contributed to the hyperpolarisation.

**Figure 5:**
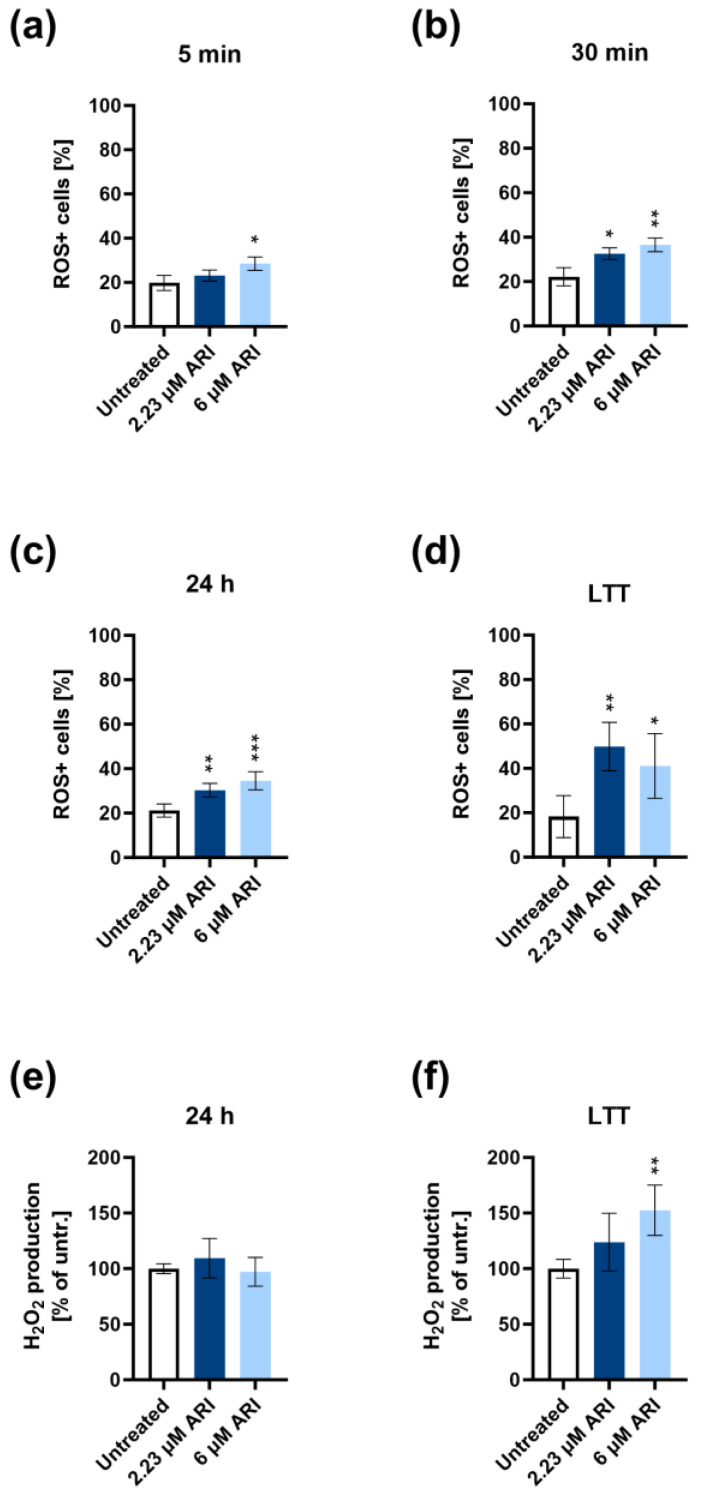
ARI increases oxidative stress in Fao cells. Percentage of ROS-positive cells (ROS+) for (**a**) 5 min (*n* = 3), (**b**) 30 min (*n* = 3), (**c**) 24 h (*n* = 4), and (**d**) long-term (*n* = 4) ARI treatments measured using microcapillary flow cytometry. H_2_O_2_ production in (**e**) 24 h (*n* = 4) and (**f**) long-term treated cells (*n* = 4). LTT: long-term treatment. Data are presented as the mean ± SD and analysed with one-way ANOVA followed by Dunnett’s test. * *p* ≤ 0.05, ** *p* ≤ 0.01, and *** *p* ≤ 0.001.

A consequence of hyperpolarisation is oxidative stress. Therefore, we checked for a ROS increase, namely, superoxide radicals (O_2_^•−^) and hydrogen peroxide (H_2_O_2_). The increased amount of superoxide radicals was first detected five minutes after the ARI addition with microcapillary cytometry (Figure 5a). The earliest statistically significant difference between the untreated and ARI-treated cells was with the higher 6 μM ARI concentration. Significantly more superoxide was formed in all ARI-treated cells compared to the untreated controls after 30 min, 24 h, and a couple of months of daily ARI treatment (Figure 5b–d). The amount of ROS tended to increase with the duration of treatment. It was the largest at the 2.23 μM ARI treatment of 4 to 8 weeks, which equals the ARI levels in a patient’s blood when the laboratory must alert the clinicians of the possibility of increased side effects.

**Figure 6:**
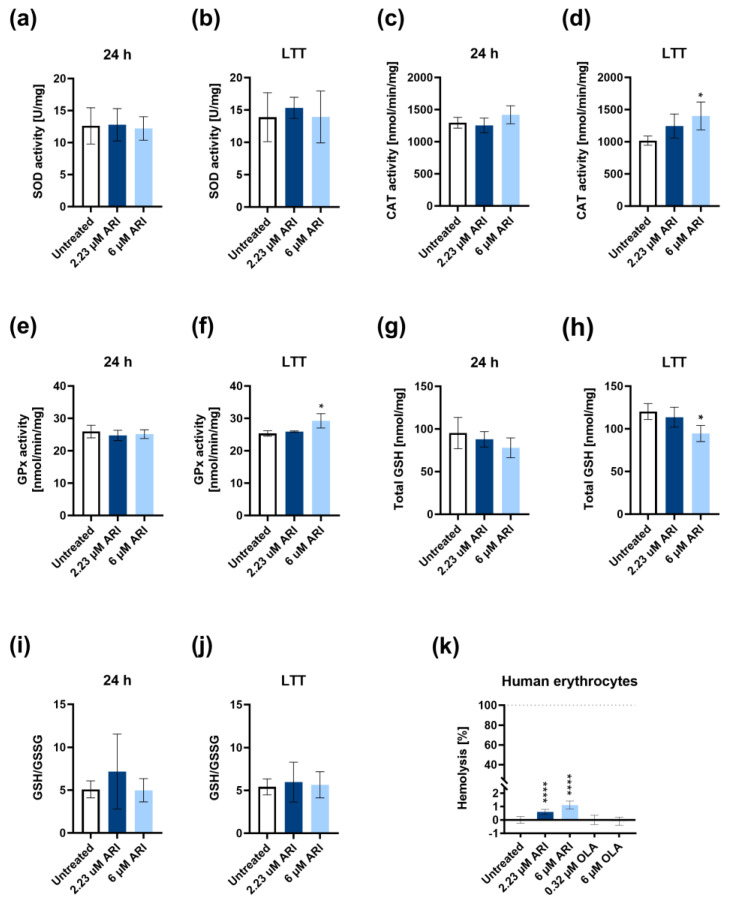
Antioxidative response in ARI-treated Fao cells and haemolysis. SOD activity in (**a**) 24 h and (**b**) long-term treated cells (*n* = 3 and 5, respectively). CAT activity in (**c**) 24 h and (**d**) long-term treated cells (*n* = 3 and 4, respectively). GPx activity in (**e**) 24 h and (**f**) long-term treated cells (*n* = 3 for each time-point). Total glutathione (GSH) content in (**g**) 24 h and (**h**) long-term treated cells (*n* = 3 and 4, respectively). The ratio of reduced to oxidised glutathione (GSH/GSSG) in (**i**) 24 h and (**j**) long-term treated cells (*n* = 3 and 4). (**k**) Haemolysis of human erythrocytes after 2 h of ARI and OLA treatments (*n* = 25). Data are presented as the mean ± SD and analysed with one-way ANOVA followed by Dunnett’s test. * *p* ≤ 0.05, **** *p* < 0.0001.

The production of H_2_O_2_ was unchanged upon the single ARI treatments over 24 h (Figure 5e). Significantly higher H_2_O_2_ production was detected exclusively after the long-term treatment with 6 μM ARI (Figure 5f). This agrees with the increased activities of the H_2_O_2_ degrading antioxidant enzymes, glutathione peroxidase (GPx), and catalase (CAT), as their activities were statistically significantly higher only in the long-term 6 μM ARI treatment (Figure 6c,f). In contrast, no increased superoxide dismutase (SOD) activity was observed upon any ARI treatment (Figure 6a,b).

The amount of total glutathione tended to be lower upon ARI treatment (Figure 6g,h). Compared to the untreated controls, it reached statistical significance only with the long-term 6 μM ARI treatment (Figure 6h). At the same time, the reduced-to-oxidised-glutathione ratios did not significantly differ in any treatment in the liver cells (Figure 6i,j). The regeneration of reduced glutathione depends on reducing equivalents of NADPH+H^+^, and so does the partition from glycolysis to pentose phosphate pathways to produce NADPH+H^+^. The effect of ARI treatment on the glutathione amount and NADPH+H^+^ levels was further assessed in human erythrocytes by haemolysis, as erythrocytes are highly sensitive to NADPH+H^+^ and glutathione levels (Figure 6k). The destruction of approximately 1% of the erythrocytes was detected only in the presence of ARI and was statistically significant compared to the untreated cells, while OLA did not have such an effect. We conclude that ARI treatment affects the availability of total and, consequently, reduced glutathione.

### Study Limitations

This study aimed to elucidate whether the observed reduction of glutathione affects erythrocytes. We used blood leftovers from healthy individuals and, apart from gender, had no information on the individuals, like in the clinical studies. The concentration of ARI equilibrated in the patients in only approximately two weeks, and the same was observed in the cell cultures [9]. As primary hepatocytes survive in cultures for only about 7 days, rat hepatoma Fao cells were used instead to enable continuous drug treatment. These cells were reported as suitable for metabolic studies [50,51,52].

## 4. Discussion

Amongst atypical antipsychotics, ARI seems to be a safer choice regarding its metabolic effects [53], although it can have severe side effects, for example, DILI [6,7]. ARI’s impact on mitochondrial metabolism and the redox state of hepatic cells, as reported in this study, can significantly contribute to these side effects. Increased mitochondrial membrane potential, or hyperpolarisation, was observed in both primary hepatocytes and Fao cells after the addition of ARI in therapeutically relevant concentrations, such as measured in the patient’s blood [47]. Mitochondrial hyperpolarisation was also observed in the PC12 neuronal model at high ARI (50 mM) and extremely high glucose concentrations (25 mM) [54]. An increase in the membrane potential immediately after ARI’s addition to the cells and isolated mitochondria implies its direct impact on the mitochondria. While there are many cases reporting transient mitochondrial hyperpolarisation [23,55], especially in experimentally induced injury and pathological processes leading to cell death [23,25,27,28,29,30], prolonged mitochondrial hyperpolarisation not leading to cell death can be beneficial for increased energy supply in wound healing by epithelial bladder cells [24] and seem widespread in cancer cells [31]. ARI treatment does not predispose the cells to cancer development. In fact, there are even reports of its cytotoxic properties in cancer cells [56]. Therefore, the long-term survival of ARI-treated cells, as described in this manuscript, implies the role of hyperpolarisation in cell stress adaptation. While increased mitochondrial production of ATP was hypothesised in bladder epithelial cells, mitochondrial hyperpolarisation in cancer cells is accompanied by increased glycolysis (Warburg effect). Increased glycolysis in ARI-treated cells compensates for decreased mitochondrial ATP production. Although less ATP is produced in glycolysis per mole of glucose compared to oxidative phosphorylation, the glucose uptake rate can increase [57]. Therefore, in the case of reduced oxidative phosphorylation, the level of glycolysis can increase to maintain a sufficient level of ATP [58,59]. The reduced dehydrogenase activity at both of the ARI concentrations used in this study [9] indicates a possible decline in the metabolic activity, which can also reduce the need for ATP synthesis. Therefore, despite the reduced mitochondrial ATP production, the total ATP content can be comparable to that in the control cells, as was measured in the ARI-treated cells.

The reduced ATP production, basal respiration, maximal respiration, reserve capacity, and nonmitochondrial oxygen consumption support an overall reduction in cellular metabolism and indicate a poor response in cases of increased energy demands [60]. There was increased proton leak across the inner mitochondrial membrane, i.e., proton transport without coupling to ATP synthesis in the ARI-treated cells. This, as well as the reduction in maximal respiration, is probably the result of the higher mitochondrial membrane potential [60], since hyperpolarisation can stimulate proton leakage [61,62].

The respiration parameters using pyruvate, glutamate, and succinate were practically unchanged in the treated and untreated cells, indicating that ARI inhibited ATP synthase in the liver cells. The decreased ATP production without affecting the maximum respiration points to a reduced ATP synthase activity [63]. No difference in the ATP synthase activity was detected by measuring its ATPase function in the ARI-treated and untreated cells. Inhibited ATP production with a simultaneously unaffected ATPase function has been reported in some neuropathy, ataxia, retinitis pigmentosa (NARP), and Leigh syndrome mutations, possibly because of disturbances in proton import into the mitochondrial matrix or defects in the coupling of proton flow through the F_0_ subunit and ATP synthesis in the F_1_ subunit [64]. Abnormalities in the mitochondrial phosphate transporter and adenine nucleotide translocase can also result in similar respirometry measurements as defects in ATP synthase [63]. The inhibition of adenine nucleotide translocase can result in mitochondrial hyperpolarisation and oxidative stress, leading to impaired mitochondrial function and presumably contributing to the development of metabolic syndrome [65]. Therefore, ARI could affect the ATP synthesis of ATP synthase or has an indirect effect through other mitochondrial proteins.

The reduced mitochondrial respiration from ARI reported here for liver cells was also observed in BV-2 microglial cells [21] and pig brain mitochondria; the latter was attributed to the reduced function of complexes I and II/III [19]. On the other hand, Scaini and coworkers [18] reported no negative effect of ARI on electron transport chain activity and ATP content in a lymphoblastoid cell line. Ota et al. [22] attributed a stimulatory effect of ARI on the Krebs cycle and complex I and decreased cytochrome c oxidase activity in the PC12 neuronal model at 50 mM ARI and 25 mM glucose, which are both extremely high compared to normal human plasma levels. The effects of ARI can be liver-specific and dependent on the experimental conditions.

The impaired electron transport chain and oxidative phosphorylation increase the production of ROS and oxidative stress. Superoxide simultaneously accumulated with the hyperpolarisation in the ARI-treated cells and remained high at all times during the ARI treatment. The formation of superoxide increased during the low respiration rate due to the greater probability of electron leakage from the electron transport chain [66]. SOD did not reduce the amount of superoxide equal to that in the ARI-treated and untreated cells, yet its activity did not increase, even after several weeks of ARI treatment. The product of SOD, H_2_O_2_, increased gradually to reach significant levels only after long-term ARI treatment. The basal activities of CAT and GPx were sufficient to maintain the rate of H_2_O_2_ formation equal to that in the untreated controls after single treatments, and the rate of H_2_O_2_ formation only increased after long-term ARI treatment with the highest ARI concentration despite the increase in CAT and GPx activities. GPx reduces H_2_O_2_ using GSH [67]. Its content was reduced in the ARI-treated cells, probably contributing to the incomplete removal of excessive H_2_O_2_. A trend of a decrease in the total GSH was observed after one day of ARI treatment, while the ratio between the GSH and GSSG was preserved, which is quite surprising given the increased oxidative stress. However, this may be a crucial factor for cell survival despite the adverse metabolic effects of ARI.

The intracellular concentration of GSH depends on a dynamic balance between the synthesis, consumption rate, and transport of the GSH [68]. GSH could be consumed into conjugated products during ARI metabolism. Bauman et al. [42] detected at least three different conjugated products of ARI in vitro. GSH consumption could additionally be increased because of protein glutathionylation, a redox modification that affects the activity of proteins and, thus, enables the regulation of cellular signalling during increased oxidative stress [68].

Erythrocytes are especially sensitive to the availability of GSH, usually seen in defects of glucose-6-phosphate dehydrogenase (G6PD), a key enzyme of the pentose phosphate pathway that indirectly maintains the level of reduced GSH. Its partial inhibition causes an increased sensitivity of erythrocytes to oxidative stress, leading to haemolytic anaemia, usually triggered by consuming drugs or food with high oxidative potential [69,70]. ARI consumption of GSH resulted in partially haemolysed human erythrocytes in our study, which agrees with four reported cases of haemolytic anaemia due to ARI [71]. Additionally, the direct inhibitory effect of ARI on the activity of G6PD purified from human erythrocytes was reported [72]. Partial G6PD inhibition slows the pentose phosphate pathway and directs glucose-6-phosphate into glycolysis. This may compensate for the ARI-induced reduction in mitochondrial ATP synthesis. The consequences of G6PD inhibition, including inhibited cell proliferation, increased oxidative stress, and enhanced glycolytic pathway, were reported in the breast cancer cell line MCF7 [73] and are consistent with our observations reported here and before, i.e., decreased proliferation [9].

## 5. Conclusions

ARI causes moderate perturbations of metabolism, possibly with one defect rescuing the other. The result of increased antioxidant enzyme activity is increased resilience to oxidative stress, which makes it an effective drug for schizophrenia in which oxidative stress is constantly present because of disease and treatment. Consistent haemolysis of 1% of human erythrocytes is an important finding that needs more testing and follow-up to resolve whether ARI can contribute to or cause haemolytic anaemia, not least for the renewed assessment by the Food and Drug Administration (FDA) and other drug regulators.

## Data Availability

Data are available from the repository of the University of Ljubljana https://repozitorij.uni-lj.si/IzpisGradiva.php?id=151979.

## Supplementary Materials

The following supporting information can be downloaded at: https://www.mdpi.com/article/10.3390/antiox12111930/s1, Figure S1: (a) Chemical structure of aripiprazole (PubChem); (b) detailed depiction of biological replicates from Figure 1f; Figure S2: ATP production in Fao cells treated with or without OLA for 24 h (a) Total cellular ATP measured with luminescent assay (*n* = 4). (b) Mitochondrial and (c) glycolytic ATP production rates were calculated from respirometry measurements (*n* = 4). (d) Glycolytic ATP production rate expressed as a percentage of overall ATP production rate. (e) oxygen consumption rate (OCR) values; (f) extracellular acidification rate (ECAR) values (*n* = 4). Data are presented as the mean ± SD and were analysed with one-way ANOVA.; Figure S3: Contribution of individual mitochondrial complexes to OCR. Data from three biological replicates (*n* = 3) are presented as the mean ± SD and were analysed with one-way ANOVA.
