# Supplementary material for: Therapeutic Dosage of Antipsychotic Drug Aripiprazole Induces Persistent Mitochondrial Hyperpolarisation, Moderate Oxidative Stress in Liver Cells, and Haemolysis
Source: Antioxidants (Basel). 2023 Oct 30;12(11):1930. doi: 10.3390/antiox12111930 (PMC10669280; doi:10.3390/antiox12111930)
Supplement: Supplementary file 1 [file antioxidants-12-01930-s001.zip › antioxidants-2652386-supplementary.pdf]

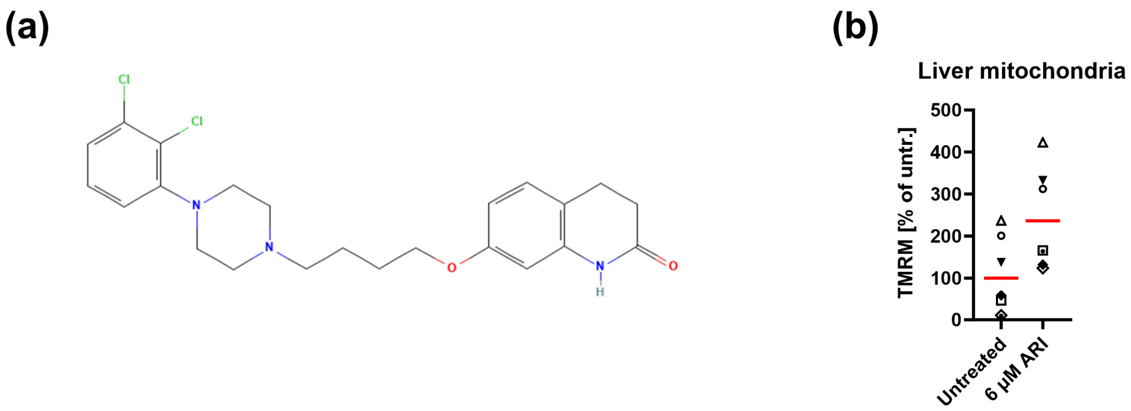

Figure S1: (a) Chemical structure of aripiprazole (PubChem); (b) detailed depiction of biological replicates from Figure 1f.

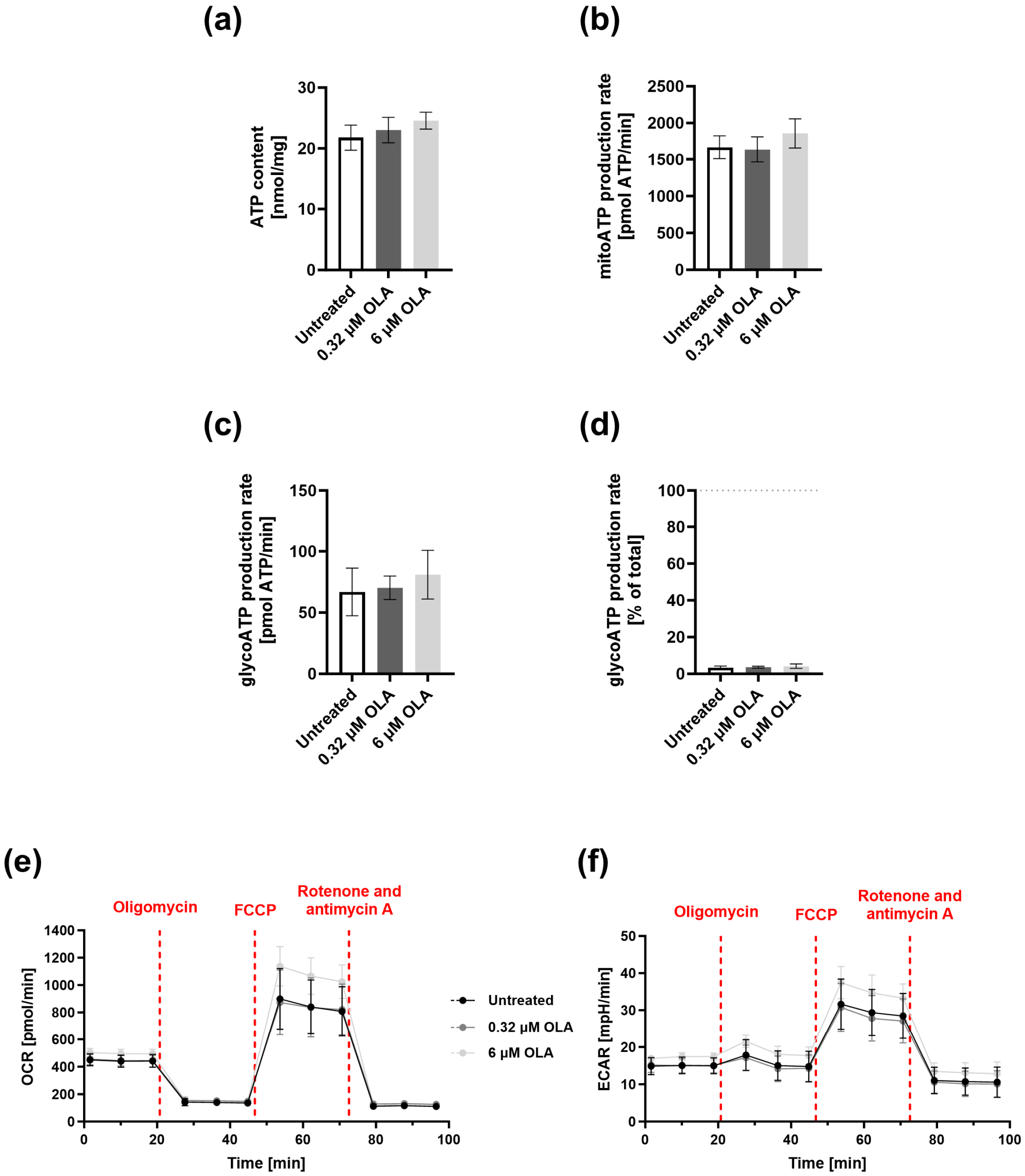

Figure S2: ATP production in Fao cells treated with or without OLA for 24 h (a) Total cellular ATP measured with luminescent assay (n = 4). (b) Mitochondrial and (c) glycolytic ATP production rates were calculated from respirometry measurements (n = 4). (d) Glycolytic ATP production rate expressed as a percentage of overall ATP production rate. (e) oxygen consumption rate (OCR) values; (f) extracellular acidification rate (ECAR) values (n = 4). Data are presented as the mean ± SD and were analysed with one-way ANOVA.

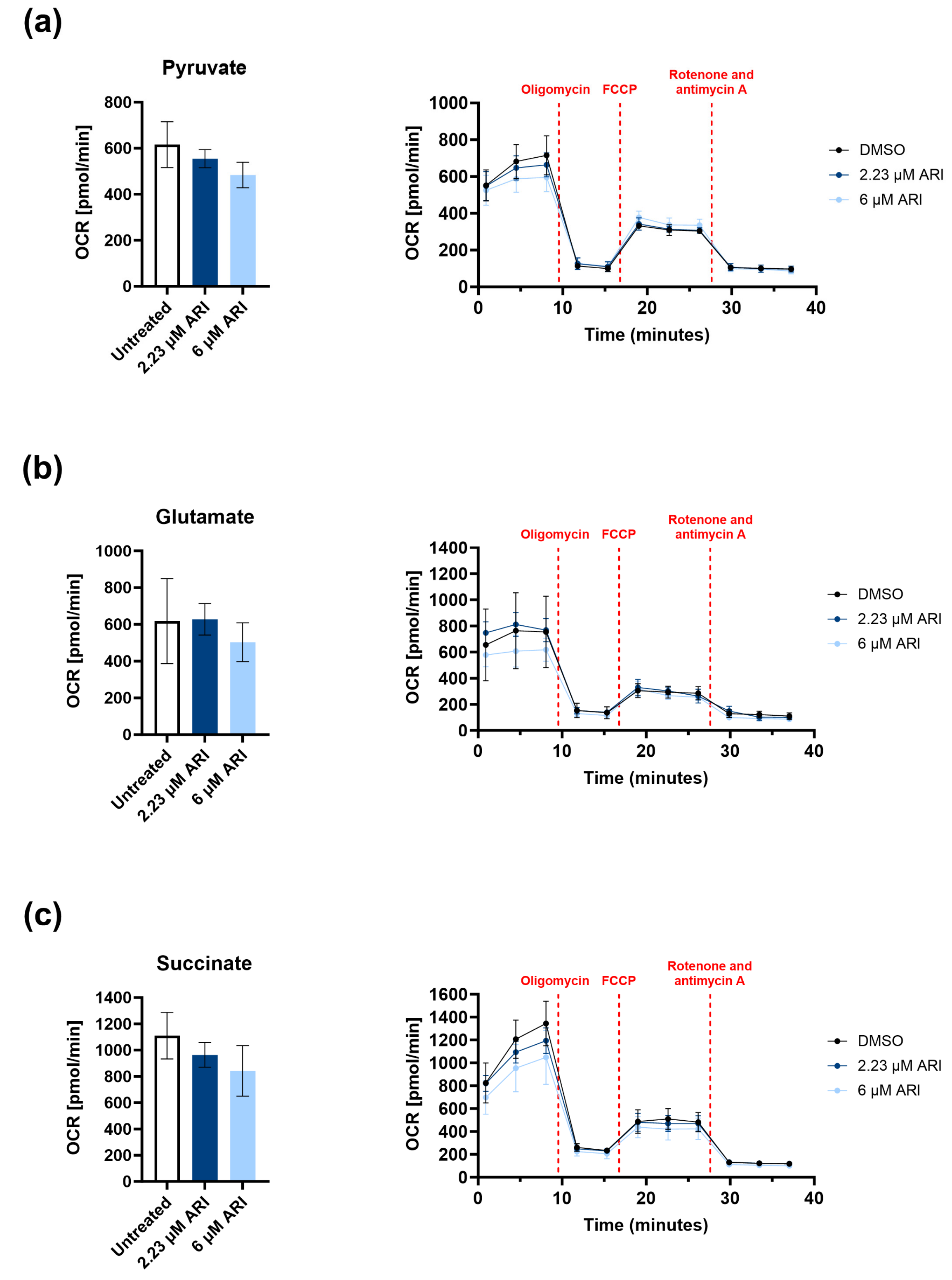

Figure S3: Contribution of individual mitochondrial complexes to OCR ($n = 3$).
